# No effect of passive stretching on neuromuscular function and maximum force-generating capacity in the antagonist muscle

**DOI:** 10.1007/s00421-021-04646-z

**Published:** 2021-03-26

**Authors:** Emiliano Cè, Giuseppe Coratella, Christian Doria, Susanna Rampichini, Marta Borrelli, Stefano Longo, Fabio Esposito

**Affiliations:** 1 Department of Biomedical Sciences for Health, Università degli Studi di Milano, Via Giuseppe Colombo 71, 20133 Milano, Italy; 2 IRCSS Galeazzi Orthopaedic Institute, Milano, Italy

**Keywords:** Supraspinal, Spinal, Peripheral, Nerve stimulation, Maximum voluntary contraction, EMG

## Abstract

**Purpose:**

The present study investigated whether or not passive stretching increases the force-generating capacity of the antagonist muscle, and the possible neuromuscular mechanisms behind.

**Methods:**

To this purpose, the neuromuscular function accompanying the force-generating capacity was assessed in 26 healthy male volunteers after passive stretching and in a control session. Before and after passive intermittent static stretching of the plantar flexors consisting of five sets × 45 s + 15 s-rest, maximum voluntary isometric contraction (MVC) and surface electromyographic root mean square (sEMG RMS) were measured in the *tibialis anterior* (the antagonist muscle). Additionally, evoked *V* wave, *H*-reflex, and *M* wave were elicited by nerve stimulation at rest and during MVC. Ankle range of motion (ROM) and plantar flexors MVC and EMG RMS were measured to check for the effectiveness of the stretching manoeuvre.

**Results:**

No change in MVC [*p* = 0.670; effect size (ES) − 0.03] and sEMG RMS/*M* wave during MVC (*p* = 0.231; ES − 0.09) was observed in the antagonist muscle after passive stretching. Similarly, no change in *V* wave (*p* = 0.531; ES 0.16), *H*-reflex at rest and during MVC (*p* = 0.656 and 0.597; ES 0.11 and 0.23, respectively) and *M* wave at rest and during MVC (*p* = 0.355 and 0.554; ES 0.04 and 0.01, respectively) was observed. An increase in ankle ROM (*p* < 0.001; ES 0.55) and a decrease in plantar flexors MVC (*p* < 0.001; ES − 1.05) and EMG RMS (*p* < 0.05; ES − 1.72 to − 0.13 in all muscles) indicated the effectiveness of stretching protocol.

**Conclusion:**

No change in the force-generating capacity and neuromuscular function of the antagonist muscle after passive stretching was observed.

## Introduction

Passive stretching is widely employed in sports and rehabilitation to improve joint range of motion (ROM). Concomitant with an increase in ROM, a reduction in force-generating capacity during maximal voluntary contractions (MVC) has often been described in the acutely stretched muscle (Avela et al. 1999; Esposito et al. 2011a; Longo et al. 2014). The increase in ROM and the reduction in the force-generating capacity were attributed to neuromuscular factors, e.g., an alteration in the afferent feedback by type-I*a*, type-II (muscle spindles) (Avela et al. 1999), type-III (mechanoreceptors), and type-IV (metabo-/nociceptors) fibres (Venturelli et al. 2017, 2019), as well as to mechanical factors, e.g., alterations in the viscoelastic properties of the muscle–tendon unit (Longo et al. 2014; Cè et al. 2015). Specifically, the neuromuscular factors may have their origin in supraspinal inhibition and reduction in spinal reflex excitability, and/or be of peripheral origin, i.e., possible impairment in the events involved in excitation–contraction coupling processes; however, their contribution to reducing contractile force-generating capability remains to be elucidated (Trajano et al. 2017; Pulverenti et al. 2020).

Recent research has focused on the possible effects of passive stretching on the antagonist muscle not directly exposed to passive stretching (Sandberg et al. 2012; Miranda et al. 2015; Wakefield and Cottrell 2015; Serefoglu et al. 2017). The rationale underpinning these studies was that the decrease in the force-generating capacity in the stretched muscle might promote the force-generating capacity in the antagonist muscle. Indeed, an increase in antagonist muscle isokinetic strength after an agonist-stretching bout was shown (Sandberg et al. 2012). Also, increased vertical jump height was reported after a hip-flexor stretching session (Wakefield and Cottrell 2015). Additionally, greater number of repetitions in seated row was observed after stretching the antagonist *pectoralis major* (Miranda et al. 2015). In contrast, no change in antagonist muscle strength was reported after passive stretching (Serefoglu et al. 2017). Should further evidence of stretch-induced increase in antagonist muscle be provided, this would be useful in both sports and rehabilitation practice. However, none of these studies investigated the possible neuromuscular mechanisms underlying the potential stretch-induced increase in strength in the antagonist muscle.

Surface electromyography (sEMG) coupled with peripheral nerve stimulation could help to estimate the possible neuromuscular changes induced by passive stretching of the antagonist muscle (Rozand et al. 2015). Particularly, the *H*-reflex overall reflects the response of the motoneuron pool to a volley from large-diameter primary muscle spindle afferents (Rozand et al. 2015). Additionally, I*a* pathways and I*b* afferents exert an influence on the *H*-reflex (McNeil et al. 2013). Moreover, the *M* wave represents the maximal compound muscle action potential (Grosprêtre et al. 2019). Remarkably, when evoking the *M* wave during MVC, the wave is followed by a reflexive response, the *V* wave (Upton et al. 1971). This response results from a collision in motor axons between the anti-dromic impulse generated by the stimulation and the descending neural drive (Upton et al. 1971; McNeil et al. 2013). This evoked response is a methodological variant of the *H*-reflex and its amplitude depends on the number of spinal motoneurons recruited and on their firing frequency during the MVC (McNeil et al. 2013). As such, the *V* wave amplitude reflects both spinal processes via reflex excitability and pre- and post-synaptic inhibition, and the level of neural drive in the descending corticospinal pathways (Balso and Cafarelli 2007). Hence, by assessing the *H*-reflex during MVC concomitantly with the *V* wave, an index of efferent spinal motor output can be retrieved (Grosprêtre et al. 2019).

To date, only one study investigated the possible stretch-induced mechanisms leading to an increase in strength in the antagonist muscle (Masugi et al. 2017). However, in this study, the antagonist muscle strength was not assessed and only spinal factors were examined, with no change in spinal excitability observed in the antagonist muscle after passive stretching (Masugi et al. 2017). This is somewhat in accordance with previous literature reporting no change in reflex excitability when passively shortening a muscle, a condition that could mimic the antagonist muscle condition while elongating the stretched muscle (Romanò and Schieppati 1987; Pinniger et al. 2001; Nordlund et al. 2004; Duclay and Martin 2005). Therefore, the aims of the present study were: (i) to examine the passive stretch-induced effects on the antagonist muscle force-generating capacity, and (ii) to deepen the possible neuromuscular changes associated with the possible change in force-generating capacity of the antagonist muscle.

## Methods

### Study design

For this cross-sectional, within-subject study, the sample size calculation was based on a previous investigation, in which the strength increase in antagonist muscle was the reference parameter (Sandberg et al. 2012), and was computed using statistical software (G-Power 3.1, Düsseldorf, Germany). Cohen’s *d*_*z*_ effect size (ES = 0.65) computed using the referenced study, a two-tail effect (*α* = 0.05), and a required power (1 − *β* = 0.80), resulted in a desired sample size of 22 participants. A total of 26 participants were recruited to overcome a drop in statistical power due to withdrawals.

### Participants

The study population was 26 healthy male volunteers [age, 24.2 (4.6) years; height, 1.76 (0.10) m; body mass 75.6 (7.5) kg, mean (SD)]. Women were excluded to avoid possible influence of the menstrual cycle on the dependent variables (Mendonca et al. 2020). The participants were recreationally active. Inclusion criteria were the elicitability to the *H*-reflex, no evident orthopaedic and/or neurological disorders; no lower-limb muscular or joint injury in the previous 6 months; and no involvement in a systematic passive stretching routine in the previous 6 months.

The local University Ethics Committee approved the study (*CE* 27/17), which was performed in accordance with the principles of the latest version of the Declaration of Helsinki. All participants gave their written, informed consent after receiving an explanation of the study purpose and design. The participants were free to withdraw from the study at any time. No dropout occurred.

### Procedures

The participants were tested at the same time of the day in a climate-controlled laboratory (temperature 20 °C ± 1 and relative humidity 50% ± 5) to minimize confounders from circadian rhythms. Plantar flexors and dorsiflexors were selected as the stretched and the antagonist muscle, respectively. Three laboratory sessions were conducted. In the first session, the participants familiarized themselves with the experimental set-up and passive stretching protocol, the MVC and the nerve stimulation procedures. Skin landmarks (moles, scars, angiomas) were mapped and the position of the angle transducers, sEMG, and stimulation electrodes were drawn on transparent sheets for accurate electrode repositioning within the same area. The second and third sessions were randomized. Out of these two, a session served as a control, in which the testing procedures were performed without any intervention in the dominant limb (kicking limb). In this session, the procedures were randomly performed in the stretched or antagonist muscle first. In the remaining session, the participants received passive stretching of the plantar flexors. The effect of stretching protocol on the antagonist muscles was assessed by dorsal flexors MVC, sEMG and the evoked responses in *tibialis anterior.* Furthermore, dorsiflexion ankle joint ROM, plantar flexors MVC, and *gastrocnemius medialis, lateralis,* and *soleus* sEMG were monitored to assess the effects of the stretching protocol. After stretching, the testing procedures were executed first on the antagonist and then on the stretched muscle, since passive stretching-induced strength loss in the stretched muscle is known to last up to 2 h after a total of 220 s of intermittent passive stretching performed on plantar flexors (Esposito et al. 2011b).

At baseline, the assessment of MVC for dorsiflexors and plantar flexors was separated by at least 5 min of passive recovery, and its order was randomized. Separate nerve stimulations were used to evoke *H*-reflex, *M* wave, and *V* wave responses in the *tibialis anterior* only. The participants performed a standardized warm-up prior to MVC and to evoke superimposed responses. Two stimulations were performed on the *tibialis anterior* for each MVC: the first at rest before (maximal response), and the second one during the plateau of MVC (superimposed response) (Grosprêtre et al. 2019). Particularly, this latter was performed because the evoked response at rest may not account for changes I*a* afferent pathways during voluntary contraction after stretching (Pulverenti et al. 2020). Hence, assessing *H*-reflexes during MVC would be more appropriate, considering the state-dependent changes affecting I*a* afferent reflex excitability and *H*-reflexes, such as decreases in homosynaptic depression and both homonymous and heteronymous I*b* inhibition, in addition to maintain motoneurons excitability (Burke et al. 1989). A rest period of 1 min was allowed before the beginning of the stretching protocol. Immediately after stretching, a single MVC was performed and two stimulations (for the antagonist muscle) were delivered at the same intensity used at baseline to elicit separately each *H*-reflex and then each *M* wave.

### Ankle range of motion

To monitor the changes in ankle joint ROM, a bi-axial electrogoniometer (mod. TSD 130A, Biopac System, Goleta, CA, USA) was utilized. The electrogoniometer was positioned with one axis on the external face of the fibula and the other on the calcaneum. The electrogoniometer signals were sent to an A/D converter (mod. UM 150, Biopac System, Goleta, CA, USA), sampled at 1000 Hz, and stored on a personal computer. The subject was prone on a medical bed. The starting ankle angle was 90°. To assess the changes in ROM, an operator slowly dorsiflexed manually the ankle joint using a visual feedback that provided her/him with angular displacement by time, so to have a possible constant velocity. The sEMG signal was checked to monitor possible muscle activation during elongation in both stretched muscles and *tibialis anterior*. During the passive stretching bout, if the sEMG signal was > 5% of that obtained during the MVC, the participant was excluded from the study and replaced with another participant to ensure the warranted statistical power (Cè et al. 2020). Three trials were performed. The maximum angle reached in each set was measured to calculate the maximum ROM.

### Maximum voluntary contraction

The MVC for the antagonist and the stretched muscle was measured with the participant prone on a medical bed, with one foot fixed by a Velcro strap to a mobile metal plate instrumented with a load cell (SM-2000 N, Interface, Crowthorne, UK). The hips and shoulders were firmly secured to the ergometer. After a standardized warm-up (10 × 2-s contractions at 50% MVC determined during familiarization), two MVCs were performed at baseline, separated by at least 3 min of passive recovery. Further trials were performed if MVC increased more than 5%. After the stretching protocol, one MVC trial was performed. The participants were instructed to push or pull as fast and hard as possible for 4 s and to focus on dorsal or plantar flexion, while avoiding any other unnecessary movement (e.g., knee flexion and hip extension/flexion) (Grosprêtre et al. 2019). Visual feedback was provided to the participants. The force signal was transmitted to an A/D converter (mod. UM 150, Biopac System, Goleta, CA, USA), sampled at a fixed sampling rate of 1000 Hz, and stored on a personal computer. The maximum force recorded was defined as the MVC and entered in the data analysis.

### sEMG recordings

**Fig. 1:**
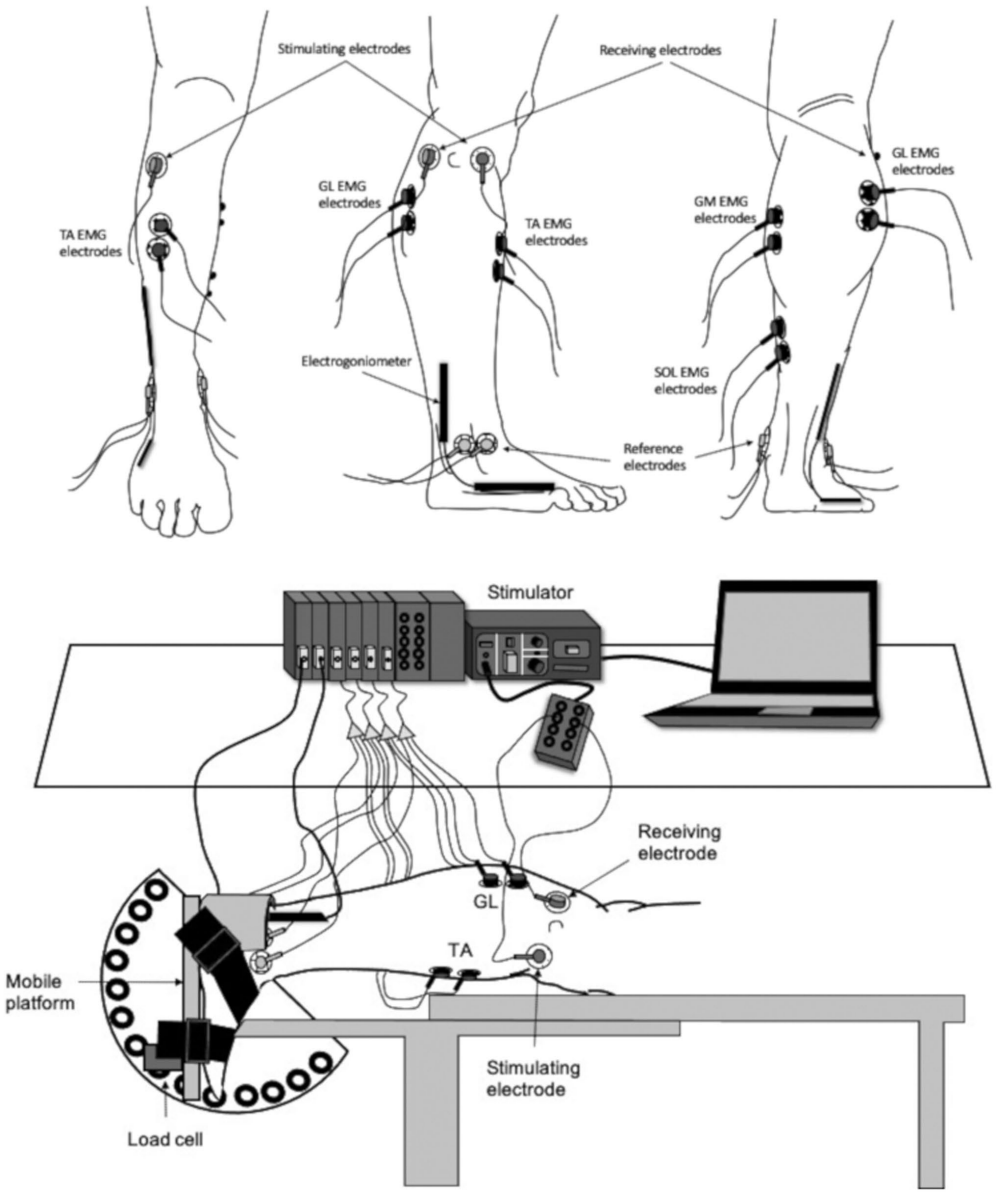
The electrodes placement and the experimental set-up are shown

Figure 1 illustrates the electrode placement and the experimental set-up. sEMG signal was recorded from the *gastrocnemius medialis, gastrocnemius lateralis, soleus*, and *tibialis anterior* muscles. The skin was shaved and then cleaned with alcohol to keep impedance low (< 5 kΩ); EMG signals were obtained using solid hydrogel rounded electrodes (mod H124SG Kendall ARBO; diameter, 10 mm; interelectrode distance, 20 mm; Kendall, Donau, Germany). The electrodes were firmly strapped to the leg and positioned 2 cm below the insertions of the gastrocnemii over the Achille’s tendon for the *soleus*, and over the mid-belly of the gastrocnemii muscles for the two gastrocnemii (Hermens et al. 2000). sEMG signal of the *tibialis anterior* was recorded with a sensor placed at 1/3 of the distance on the line between the fibula and the tip of the medial malleolus (Hermens et al. 2000). Following visual inspection of the sEMG signal, the electrodes were eventually replaced should a crosstalk contamination of the sEMG signal of the plantar flexors occur (Mendonca et al. 2020). The surface EMG signal was detected during MVC and nerve stimulation and acquired by a multichannel amplifier at a sampling rate of 2048 Hz (mod. DA100 UM 150, Biopac, Biopac System Inc.; input impedance > 90 MΩ; CMRR > 96 dB), amplified (gain × 1000) and filtered (filter type: IV order Butterworth filter; bandwidth 10–500 Hz) for further analysis.

### Nerve stimulation

Single rectangular pulses (10 mA–1 A, 100–150 V square wave pulse of 1 ms in width) were delivered to the deep peroneal nerve for dorsiflexor muscle stimulation. A self-adhesive Ag–AgCl cathode (diameter, 8 mm) was placed under the fibula head and an anode (5 × 10 cm, Medicompex SA, Ecublens, Switzerland) was placed in the posterior head of the fibula (Fig. 1). The electrodes were connected to a high-voltage stimulator (Digitimer Stimulator, Model DS7AH, Hertfordshire, UK). The nerve stimulation and sEMG signal was recorded with a multichannel amplifier at a sampling rate of 2048 Hz (Mod. UM 150, Biopac System, Goleta, CA, USA).

The stimulation intensity started from 5 mA and was progressively increased by 2 mA increments in the *tibialis anterior* to detect the maximal *H*-reflex (*H*_max_). Thereafter, the stimulation intensity was increased by 5 mA increments to detect the maximal *M* wave (*M*_max_) until *M*_max_ no longer increased. This last stimulation intensity was then increased by 20% to ensure supramaximal stimulation and used to record the maximal *M* wave (*M*_max_). At baseline, four stimulations were performed at each intensity to build the whole recruitment curve to determine the optimal stimulation for *H*_max_ and *M*_max_ both in the control and stretching session (Grosprêtre et al. 2019) before starting the passive stretching protocol. More in detail, the procedures to build such a recruitment curve were an inter-stimulus interval of 10 s; the start of the curve was the lowest current which was sufficient to evoke an *H*-reflex. The same within-subject stimulation intensities were then used to evoke the superimposed (*H*_sup_ and *M*_sup_) for the baseline and for the post-stretching assessment (Grosprêtre et al. 2019).

**Fig. 2:**
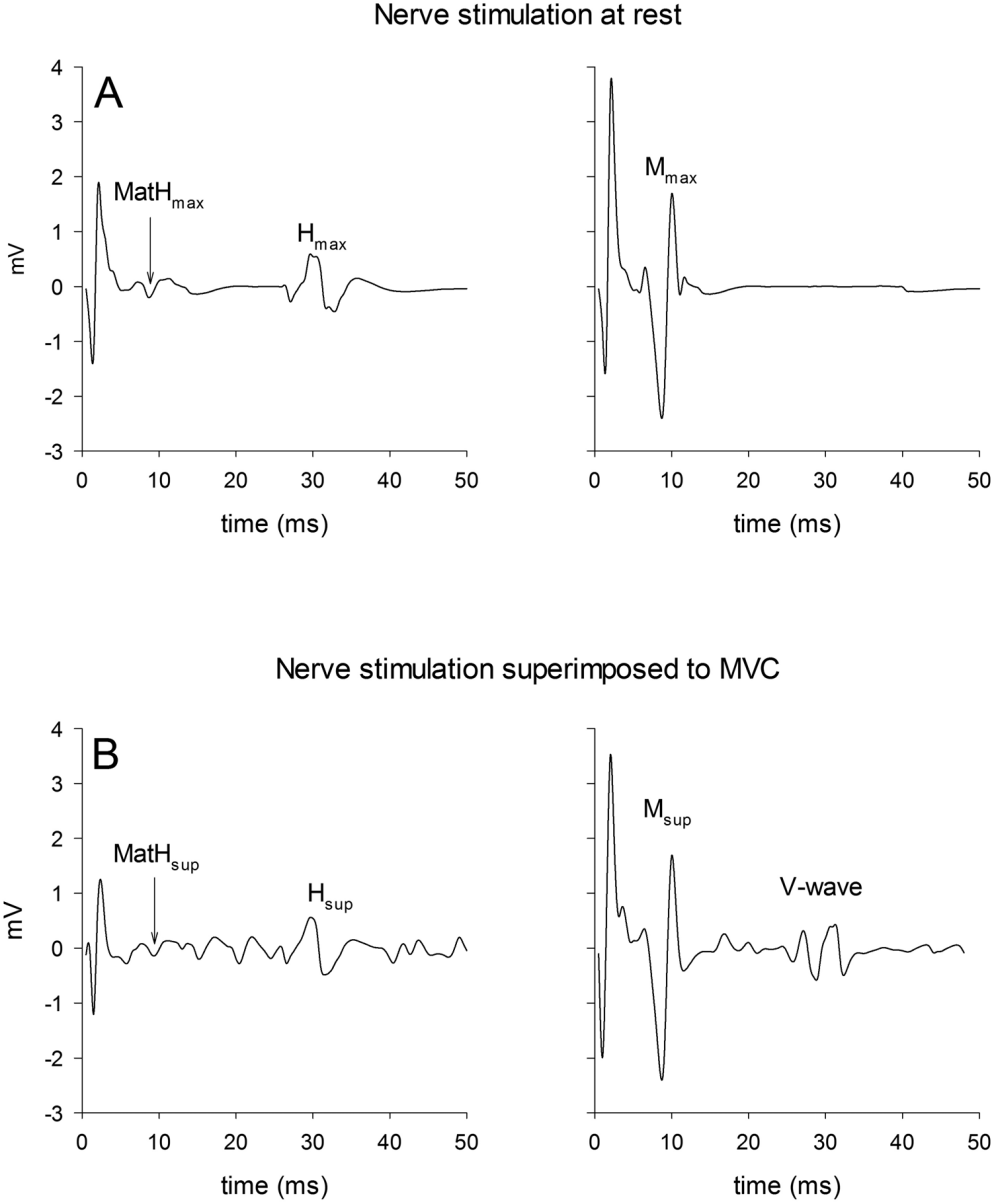
A typical sEMG signal with electric stimulation is shown at rest (**a**) and during MVC (**b**). The *y*-axis intercept represents the time-course starting from the electrical stimulation artifact

A dedicated software (Mod. Stim 100C, Biopac System Goleta, CA, USA) enabled us to have the same stimulation order (first *H*-reflex, then *M* wave) at a fixed temporal distance (500 ms) for each stimulation. Responses at rest were assessed without prior muscle activity at an interval of 10 s. Superimposed responses were manually triggered after the MVC plateau was attained. The *V* wave was recorded during the superimposed *M* wave stimulation, at the same time delay between stimulation onset and the occurrence of the *H*-reflex (Grospretre and Martin 2014). A typical signal recording from a representative participant is shown in Fig. 2.

### Data analysis

The researchers who analysed the data were blinded to the testing sessions (control or stretching). During the MVCs, the sEMG signal was analysed in the time domain within a 1-s period of the MVC plateau without any stimulation, and it was assessed by visual inspection. The sEMG root mean square (sEMG RMS) was calculated in consecutive 250-ms time windows, averaged, and then entered in the data analysis. The *M*_max_ and *M*_sup_ were entered into the data analysis. The average peak-to-peak amplitudes of the evoked responses were calculated and normalized to the maximal *M* wave evoked in the same condition. Thus, *H*_max_/*M*_max_, *H*_sup_/*M*_sup_, *V*/*M*_sup_, together with sEMG RMS/*M*_sup_ were defined as dependent variables and were compared pre- and post-stretching. The normalization of the *H*-reflex to the *M* wave was done to provide information about the proportion of the motoneuron pool being activated in the *H*-reflex (Knikou 2008). As *V* wave involves both spinal and supraspinal mechanisms, the comparison of the changes in *H*_sup_/*M*_sup_ and *V*/*M*_sup_ was proposed as a tool to estimate the relative contribution of both levels to *V* wave changes (Gondin et al. 2006). The *M* wave accompanying the maximal *H*-reflex was measured and normalized to the corresponding maximal *M* wave (MatH_max_/*M*_max_ and MatH_sup_/*M*_sup_) (Grosprêtre et al. 2019) to ensure the consistency of the stimulus condition during the whole experiment (Grosprêtre and Martin 2012).

### Stretching protocol

During the passive stretching protocol, the participants remained prone on the same medical bed and with the same ergometer used for the testing procedures. An operator dorsiflexed the ankle of the stretched limb until 90% of maximal discomfort, according to the subjective response for each participant. Particularly, a 0–10 visual analogue scale was used at this purpose, spanning from no-discomfort to maximal discomfort (Venturelli et al. 2019). The stretching intensity was kept constant by mean of a constant force output exerted by the operator. The force output between the passively stretched leg and the operator’s arms was recorded during the protocol by a load cell (SM-2000 N, Interface, Crowthorne, UK) (Venturelli et al. 2019). Specifically, the load cell was positioned 5 cm above the metatarsus of the passively stretched limb and an operator pushed perpendicularly the load cell to stretch plantar flexors. To minimize possible muscle reflex activity, muscle elongation was reached in 6 s and maintained for 45 s (Esposito et al. 2011a, b). Five sets with 15-s intervals of passive recovery were performed for a total duration of 225 s. The sEMG signal was checked during passive stretching to monitor possible muscle activation during elongation in both stretched and *tibialis anterior*. If the sEMG signal during passive stretching was > 5% of that obtained during MVC, the participant was excluded from the study and replaced with another one to ensure statistical power (Cè et al. 2020). In the control session, the participants lay prone as relaxed as possible with the ankle at a neutral angle (90°) for an equivalent duration.

### Statistical analysis

Statistical analysis was performed using a statistical software package (IBM SPSS Statistics 22, Armonk, NY, USA). The Shapiro–Wilk’s test was applied to check for normal distribution of the sampling. To determine inter-day reliability, the intra-class correlation coefficient (ICC) and the standard error of the measurement (SEM %) were calculated using the baseline values recorded during the second and third sessions. The ICC was interpreted as follows: very high (≥ 0.90); high (0.89–0.70); moderate (0.69–0.50). The minimal detectable change with a 95% confidence interval (MDC 95%) was used to detect the sensitivity of the intervention. The pre–post differences in MVC of the stretched and the antagonist muscle compared to their respective control were calculated by time (2 levels: pre and post) × condition (2 levels: stretching and control) repeated-measures analysis of variance (ANOVA). The pre–post differences in sEMG RMS/*M*_sup_ in the *gastrocnemius medialis, gastrocnemius lateralis, soleus*, and *tibialis anterior* compared to their respective control were calculated by separate time × condition repeated-measures ANOVA. The pre–post differences in all evoked responses compared to the control were calculated by time × condition repeated-measures ANOVA. Multiple comparisons were performed using Bonferroni’s correction. Significance was set at *α* < 0.05. Unless otherwise stated, descriptive statistics are presented as mean (± SD). The changes are reported as percentage change with 95% confidence interval (95% CI). Cohen’s *d* effect size (ES) was calculated and interpreted as follows: trivial (0.00–0.19); small (0.20–0.59); moderate (0.60–1.19); large (1.20–1.99); very large (≥ 2.00) (Hopkins et al. 2009)*.* The 95% CI of the ES is reported.

## Results

Table 1 presents the reliability values. All parameters showed *very high* reliability, with a MDC 95% from 6.6 to 19.9%.

**Table 1:** The reliability values are shown for each dependent parameter

Nerve stimulation parameters (mV)	Stretching m (SD)	Control m (SD)	ICC	SEM %	MDC 95%
H_max_	0.711 (0.372)	0.709 (0.375)	0.902	16.4	45.5
H_sup_	0.715 (0.355)	0.722 (0.371)	0.875	17.5	48.4
M_max_	3.600 (1.979)	3.576 (2.017)	0.887	18.5	51.4
M_sup_	3.714 (1.964)	3.684 (1.960)	0.906	16.3	45.1
V	0.602 (0.295)	0.589 (0.301)	0.903	15.4	42.8

No change in the stretched muscle was observed during the control session. After passive stretching, the ankle joint ROM of the stretched muscle increased from 21.9 (5.3)° to 24.6 (4.4)°, *p* < 0.001, ES 0.55, 95% CI 0.01/1.10; MVC decreased from 150.1 (11.1) N to 138.6 (10.6) N, *p* < 0.001, ES − 1.05 (− 1.61/− 0.46); in the *gastrocnemius medialis*, *gastrocnemius lateralis*, and *soleus* the sEMG RMS decreased [from 0.37 (0.07) to 0.29 (0.07) mV, 0.38 (0.08) to 0.30 (0.06) mV and 0.32 (0.05) to 0.28 (0.05) mV, respectively; *p* < 0.05, ES from − 1.72 to − 0.13).

**Fig. 3:**
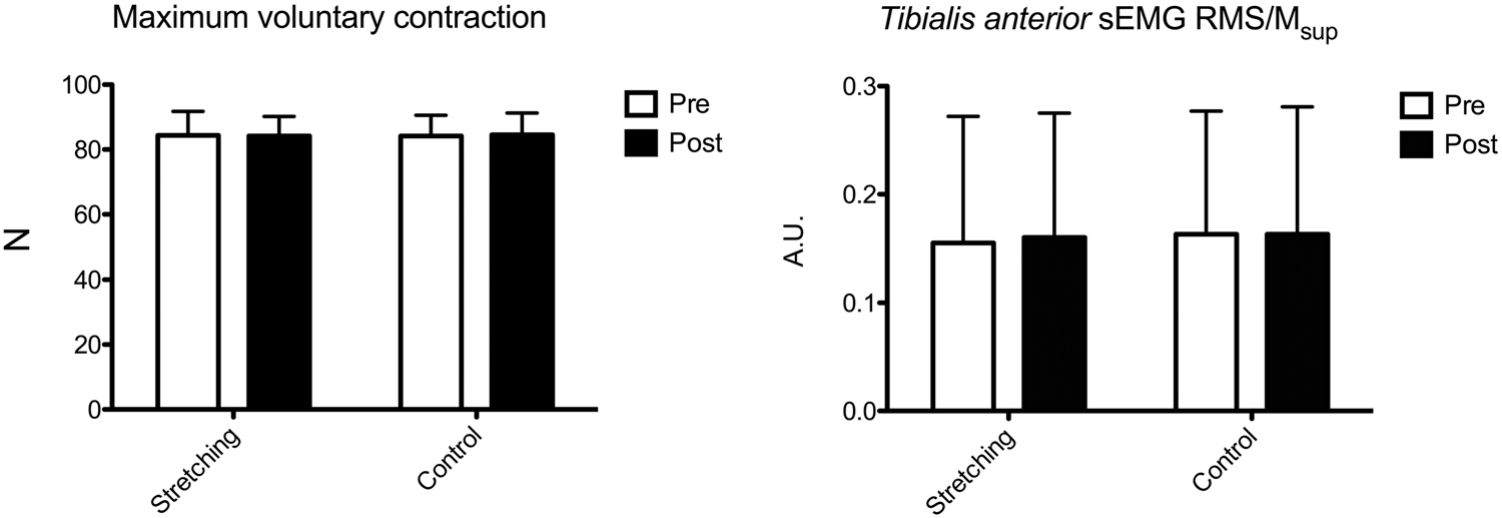
The changes in maximum voluntary contraction and sEMG RMS/*M*_sup_ for the antagonist muscle during either passive stretching or control session are shown. No difference after the stretching bout was observed

No change was observed in the antagonist muscle during the control session. Figure 3 presents the changes in MVC and sEMG RMS/*M*_sup_ of the *tibialis anterior* of the antagonist muscle. No two-way interaction was found for MVC (*p* = 0.713) and *tibialis anterior* sEMG RMS/*M*_sup_ (*p* = 0.610), and no change in MVC (*p* = 0.670, ES − 0.03, − 0.66 to 0.61) and *tibialis anterior* sEMG RMS/*M*_sup_ (*p* = 0.231, ES − 0.09, − 0.45 to 0.64) after passive stretching was recorded. Table 2 presents the changes in the neural parameters. No interaction was found for *H*_max_/*M*_max_ (*p* = 0.961), *H*_sup_/*M*_sup_ (*p* = 0.541), *M*_max_ (*p* = 0.481), *M*_sup_ (*p* = 0.510), *V*/*M*_sup_ (*p* = 0.568), MatH_max_/*M*_max_ (*p* = 0.309) and MatH_sup_/*M*_sup_ (*p* = 0.459). No change in any evoked response was observed.

**Table 2:** The pre- and post-stretching values of the neural dependent parameters for the antagonist muscle are reported as mean (SD). Changes are reported as effect size with 95% confidence interval. No change was observed

	Pre	Post	*p* value	ES (95% CI)
*V*/*M*_sup_	0.16 (0.05)	0.17 (0.07)	0.531	0.16 (− 0.40/0.73)
*H*_max_/*M*_max_	0.21 (0.08)	0.22 (0.10)	0.656	0.11 (− 0.44/0.65)
*H*_sup_/*M*_sup_	0.21 (0.08)	0.23 (0.09)	0.597	0.23 (− 0.31/0.78)
*M*_max_ (mV)	3.60 (1.98)	3.67 (1.93)	0.355	0.04 (− 0.51/0.58)
*M*_sup_ (mV)	3.71 (1.96)	3.73 (1.94)	0.554	0.01 (− 0.53/0.55)
MatH_max_/*M*_max_	0.08 (0.03)	0.09 (0.01)	0.284	0.11 (− 0.42/0.67)
MatH_sup_/*M*_sup_	0.09 (0.02)	0.10 (0.03)	0.682	0.09 (− 0.45/0.64)

## Discussion

The current study was conceived to determine whether or not (i) passive stretching increased the force-generating capacity of the antagonist muscle and (ii) which neuromuscular pathways possibly lay behind it. We observed no increase in MVC in the antagonist muscle, no change in sEMG RMS/*M*_sup_, and no change in the *H*-reflex/*M* wave ratios, *M* wave and *V*/*M*_sup_. As such, passive stretching did not increase the force-generating capacity of the antagonist muscle, nor induced any neuromuscular change.

Some preliminary considerations are needed to interpret the results properly. To check for the effectiveness of the stretching protocol, we measured the ankle joint ROM, and the MVC and sEMG RMS in the stretched muscle. As expected, the ankle ROM was increased, whereas the plantar flexors MVC and sEMG RMS were decreased, in line with previous studies (Esposito et al. 2011a, b,^ 2020^; Cè et al. 2015). A previous review highlighted that the minimal stretching dose that affects both ROM, MVC and sEMG RMS in the stretched muscle is a total of 60 s (Trajano et al. 2017). Moreover, the stretching modality seems also to influence the output, since the intermittent protocol appeared to reduce strength and muscle excitation more than a continuous protocol, matched for the total duration (Trajano et al. 2014). The strength loss and reduced excitation seem mainly to be affected by impaired central rather than peripheral mechanisms (Cè et al. 2020), with a possible contribution of a loss in spinal activity (Trajano et al. 2017). However, the present procedures do not allow further comparisons with the literature. The use of a control condition and the *high* to *very high* reliability values indicate that the lack of changes in the antagonist muscle are unlikely attributable to high variability in the recorded signals. Lastly, the small *M* waves (MatH) accompanying these reflexes were used to ensure the consistency of stimulus condition during the whole experiment (Grosprêtre and Martin 2012). As such, similar MatH/*M*_max_ indicated that the recorded *H*-reflex lay within the same portion of the recruitment curve, with stable nerve stimulation conditions (Grosprêtre et al. 2019).

The previous studies investigating the changes in the force-generating capacity of the antagonist muscle, measured muscle strength using non-isometric tests, making direct comparison with the literature challenging. Knee extensors isokinetic peak torque at 300° s^−1^ was increased in the antagonist non-stretched muscle after a stretching bout performed on the knee flexors (Sandberg et al. 2012). Similarly, gains in maximum power and countermovement jump height were reported after hip flexors were stretched (Sandberg et al. 2012; Wakefield and Cottrell 2015). Moreover, greater number of dynamic repetitions was observed in seated row after stretching the antagonist *pectoralis major* (Miranda et al. 2015). In contrast, when the antagonist muscle force-generating capacity was measured at a lower angular velocity (e.g., 60° s^−1^), no gain in torque was found in both knee extensors and flexors (Sandberg et al. 2012; Serefoglu et al. 2017). This suggests that the stretch-induced effects on the antagonist muscle are perhaps more visible when the force-generating capacity is assessed at high velocity or during explosive tasks. However, using rapid strength exertion to measure the muscle force-generating capacity would have strongly limited the possibility to assess the neuromuscular contribution separately.

As hypothesized, no change in spinal and peripheral mechanisms contribution was observed in the antagonist muscle, given the lack of change in the H/M ratios. Interestingly, spinal reflex excitability in the *tibialis anterior* was decreased during but not after passive stretching of the plantar flexors (Masugi et al. 2017). The researchers argued that muscle spindle activation during passive stretching inhibits the motor neurons in both the stretched and the ipsilateral non-stretched muscle via the heteronymous I*a* connection (Masugi et al. 2017). The mechanism seems to disappear, however, when the stretched muscle returns to baseline length (Masugi et al. 2017). This is in line with previous studies reporting no change in reflex excitability when a muscle is passively shortened, which is somehow similar to the current antagonist muscle (Romanò and Schieppati 1987; Pinniger et al. 2001; Nordlund et al. 2004; Duclay and Martin 2005). To deepen the spinal mechanisms mediating the agonist–antagonist relationship, evoking the *H*-reflex with a prior stimulation of the antagonist nerve may result in the D1 presynaptic inhibition, i.e., the presynaptic inhibition of *H*-reflex mediated by I*a* afferent of the antagonist muscle, reciprocal inhibition or I*b*-afferent (Baudry et al. 2011). Similarly, the lack of change in the *M* wave revealed that no change in neuromuscular transmission occurred (Rozand et al. 2015). Since the peripheral contribution to the force-generating capacity was demonstrated not to occur in the stretched muscle (Trajano et al. 2017; Cè et al. 2020), our findings for the antagonist muscle were actually expected.

No change in the *V*/*M*_sup_ occurred in the antagonist muscle. During MVC, the *V* wave amplitude should reflect the level of the efferent drive transmitted by the motoneurons (Aagaard et al. 2002). As such, an increase in *V*/*M*_sup_ without any change in *H*_sup_/*M*_sup_ would point out greater motoneuron recruitment or firing rate, which may indicate greater supraspinal contribution to the motoneuron pool (Aagaard et al. 2002). However, the modulation of this evoked response is not necessarily due to supraspinal changes. Increases in *V* wave could result from anything that increases motoneurons recruitment or firing, including a change of intrinsic properties of motoneurons, reduction of presynaptic inhibition of I*a*-afferents and/or even post-synaptic inhibition of spinal motoneurons (e.g. Renshaw inhibition) (McNeil et al. 2013). Notwithstanding, the motoneuron firing rate is an index of both the supraspinal input to the motoneuron and of the response to all inputs to the motoneuron, making difficult a clear identification of the origin of a possible change in *V* wave amplitude (McNeil et al. 2013). Based on the unchanged *V*/*M*_sup_ and the concomitant unchanged *H*_max_/*M*_max_ and *H*_sup_/*M*_sup_, it may be hypothesized that the activity of both the cortical (Pulverenti et al. 2020) and subcortical (i.e., cerebellum, basal ganglia, and thalamus nuclei) (Wiesendanger and Wiesendanger 1985) pathways towards the antagonist muscle did not change after passive stretching. Since no study has previously used a similar design, a direct comparison with the literature cannot be made and further elucidations are needed.

The present study has several limitations. First, supraspinal mechanisms can be investigated using various different techniques (e.g., transcranial magnetic stimulation), which would provide further information at the cortical level. Second, *H*-reflexes and *V* wave are products of small proportion of motoneurons, so that undetectable changes are still possible (McNeil et al. 2013). Third, the present outcomes are specific to the stretched/antagonist muscles selected and cannot be extended to other muscles. Fourth, the passive stretching protocol may have led to these specific results, while different stretching modalities (e.g., dynamic stretching) and duration may produce different outcomes. Lastly, the force-generating capacity was measured isometrically. We acknowledge that dynamic strength measurements might lead to different findings, although the neuromuscular contribution is not easy to assess dynamically.

In conclusion, we found no change in the force-generating capacity in the antagonist muscle after passive stretching. Additionally, no change in sEMG amplitude was observed. Lastly, no change in neuromuscular function was retrieved.

## Conclusion

Previous studies have shown an increase in fast dynamic strength and power tasks in the antagonist non-stretched muscle after a passive stretching manoeuvre (Sandberg et al. 2012; Serefoglu et al. 2017). The present findings do not support this trend, so stretching a muscle should be not used to increase the antagonist strength if this is exerted at slow or null velocity. Our findings question the notion that passive stretching enhances the isometric force of the antagonist muscle.

## Data Availability

The data that support the findings of this study are available from the corresponding author upon reasonable request.

## Abbreviations

*H*_max_: Maximal *H*-reflex
*H*_sup_: Superimposed *H*-reflex
*M*_max_: Maximal *M* wave
*M*_sup_: Superimposed *M* wave
MVC: Maximum voluntary contraction
RMS: Root mean square
ROM: Range of motion
sEMG: Surface electromyography
